# Age Also Matters for Gerontologists: Personal and Professional Perspectives on Growing Older

**DOI:** 10.1093/geroni/igaa057.2324

**Published:** 2020-12-16

**Authors:** Phillip Clark, Margaret Perkinson

## Abstract

Gerontology is a unique field of scientific inquiry, because it embodies both professional and personal dimensions of experience and poses questions for its researchers. How does our work help us understand our own personal experience of aging? How does the reality of growing older change our teaching and research? As gerontologists, we embody two narratives of the aging experience, one academic and professional (with its dependence on theory and scientific research), the other intimately personal (with its own lived experience and practical insight acquired over the life course). How this dynamic unfolds is as personal as each of us as individuals, and embodies our own disciplinary backgrounds; yet collectively it has implications for how we approach an understanding of what it means to grow old. This symposium explores different facets of this dynamic from four perspectives of different individuals and differing disciplines. The first paper assesses the limitations of both quantitative and qualitative research paradigms in revealing the deeply idiosyncratic nature of personal aging. The second develops the metaphor of “double agent of aging” to characterize the two narratives of professional and personal aging. The third uses Erikson’s theory of psychosocial development to weave together the professional, practical, and personal dimensions of gerontology. Finally, the last develops the metaphor of arcs and stages in conceptualizing a gerontological career. The symposium concludes with recommendations for the integration of theoretical, practical, and personal insights into teaching, research, and service in a way that embraces, enhances, and extends the field of gerontology.

